# Clinicopathological significance of core 3 *O*-glycan synthetic enzyme, β1,3-*N*-acetylglucosaminyltransferase 6 in pancreatic ductal adenocarcinoma

**DOI:** 10.1371/journal.pone.0242851

**Published:** 2020-11-30

**Authors:** Noriteru Doi, Yoshinori Ino, Kiyohiko Angata, Kazuaki Shimada, Hisashi Narimatsu, Nobuyoshi Hiraoka

**Affiliations:** 1 Division of Molecular Pathology, National Cancer Center Research Institute, Tokyo, Japan; 2 Department of Analytical Pathology, National Cancer Center Research Institute, Tokyo, Japan; 3 Department of Life Science and Biotechnology, The Glycoscience and Glycotechnology Research Group, Biotechnology Research Institute for Drug Discovery, National Institute of Advanced Industrial Science and Technology, Tsukuba, Japan; 4 Hepatobiliary and Pancreatic Surgery Division, National Cancer Center Hospital, Tokyo, Japan; 5 Division of Pathology and Clinical Laboratories, National Cancer Center Hospital, Tokyo, Japan; Centro Nacional de Investigaciones Oncologicas, SPAIN

## Abstract

Mucin-type *O*-glycans are involved in cancer initiation and progression, although details of their biological and clinicopathological roles remain unclear. The aim of this study was to investigate the clinicopathological significance of β1,3-*N*-acetylglucosaminyltransferase 6 (β3Gn-T6), an essential enzyme for the synthesis of core 3 *O*-glycan and several other *O*-glycans in pancreatic ductal adenocarcinoma (PDAC). We performed immunohistochemical and lectin-histochemical analyses to detect the expression of β3Gn-T6 and several *O*-glycans in 156 cases of PDAC with pancreatic intraepithelial neoplasias (PanINs) and corresponding normal tissue samples. The T antigen, Tn antigen, sialyl Lewis X (sLeX) antigen, and sLeX on core 2 *O*-glycan were more highly expressed in PDAC cells than in normal pancreatic duct epithelial cells (NPDEs). Conversely, the expression of 6-sulfo *N*-acetyllactosamine on extended core 1 *O*-glycan was found in NPDEs and was low in PDAC cells. These glycan expression levels were not associated with patient outcomes. β3Gn-T6 was expressed in ~20% of PDAC cases and 30–40% of PanINs but not in NPDEs. Higher expression of β3Gn-T6 was found in PDAC cells in more differentiated adenocarcinoma cases showing significantly longer disease-free survival in both univariate and multivariate analyses. In addition, the expression of β3Gn-T6 in PDAC cells and PanINs significantly correlated with the expression of MUC5AC in these cells, suggesting that β3Gn-T6 expression is related to cellular differentiation status of the gastric foveolar phenotype. Thus, it is likely that β3Gn-T6 expression in PDAC cells is a favorable prognostic factor in PDAC patients, and that the expression of β3Gn-T6 correlates with the gastric foveolar phenotype in pancreatic carcinogenesis.

## Introduction

Mucin-type *O*-glycans play roles in various biological functions, including lymphocyte homing and gastric mucosal defense against *Helicobacter pylori* [1–3]. Cancer cells express unique and characteristic glycan structures [4], some of which are involved in cancer initiation, progression, and metastasis, mainly through cellular recognition and/or cell adhesion [5]. Although these unique characteristics have the potential to be used for diagnostic and therapeutic research and development, limited information is currently available regarding the biological roles and clinicopathological significance of *O*-glycans in cancer.

**Fig 1:**
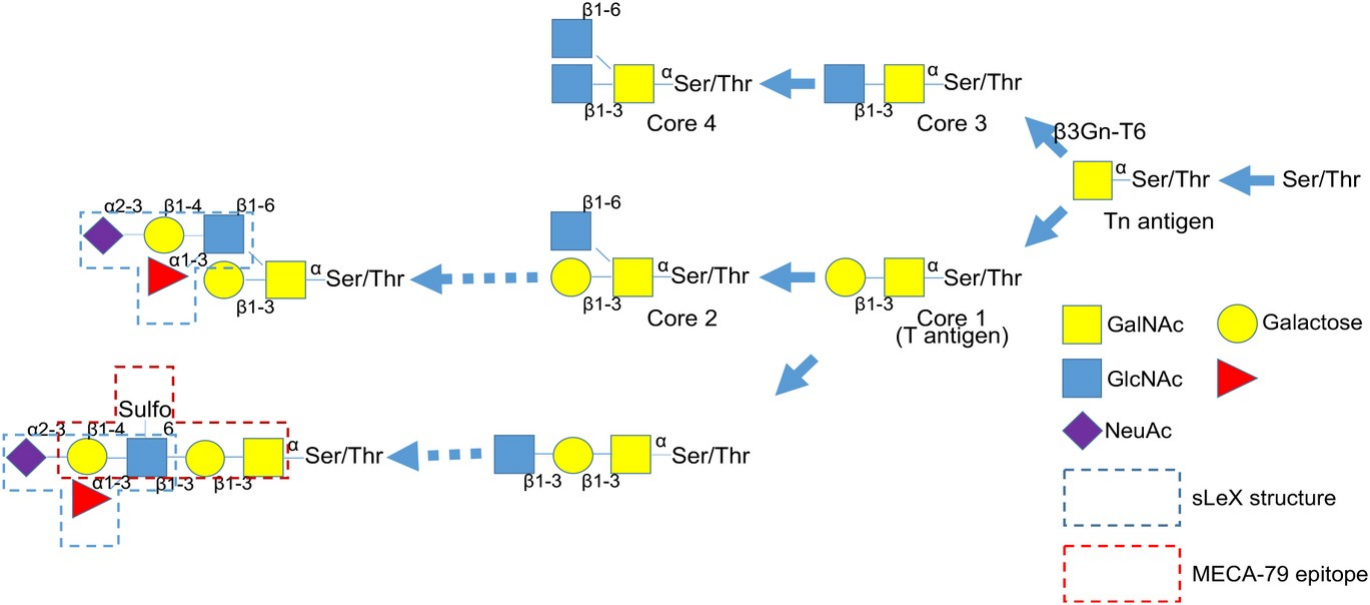
Biosynthetic pathways of mucin-type *O*-glycans. β3Gn-T6 is the only core 3 synthetic enzyme. GalNAc: *N*-Acetylgalactosamine, GlcNAc: *N*-Acetylglucosamine, NeuAc: *N*-Acetylneuraminic acid.

Glandular epithelial cells produce mucin consisting of core proteins and abundant *O*-linked glycans [6]. The synthesis of *O*-glycans is initiated by the addition of *N*-acetylgalactosamine to Ser/Thr to form the Tn antigen (Fig 1). Based on the Tn antigen, core 1 (T antigen) or core 3 structures are formed, which are then branched to give rise to core 2 or core 4 structures, sequentially. These core structures can be further extended thus resulting in complex glycans, such as several blood type antigens (Fig 1). It has been reported that both core and peripheral modified glycans are expressed specifically in some types of cancer and are related to biological characteristics of the cancer cells, thereby representing tumor markers and prognostic markers [7–10].

Pancreatic ductal adenocarcinoma (PDAC) is a highly malignant disease [11]. Despite advances in diagnosis and treatments, the 5-year survival rate is less than 10% in PDAC [12]. To improve the patient outcomes, we have to understand PDAC more deeply. PDAC is known to express the Tn antigen and its sialylated derivative, the STn antigen [13]. These truncated *O*-glycans in PDAC cells are associated with aggressive characteristics [14, 15]. Sialyl Lewis A (sLeA), alternatively called CA19-9, and sialyl Lewis X (sLeX) are reported to be unfavorable prognostic factors in PDAC [15, 16]. Although this has been previously demonstrated by studies involving cell lines or animal models, only a few reports have addressed the clinicopathological and biological roles of glycans using human clinical samples. In addition, these two unique glycans, 6-sulfo *N*-acetyllactosamine on extended core 1 *O*-glycan detected by antibody MECA-79 [3, 17], and sLeX on core 2 *O*-glycan detected by antibody NCC-ST-439 (ST-439) [18, 19] have not yet been evaluated in PDAC. The presence of MECA-79 antigen is used as a marker of high endothelial venules of lymph nodes and the antigen is a part of the glycan structure of an L-selectin ligand [3], and is expressed in pancreatic ductal epithelial cells [20].

The presence of core 3 *O*-glycan that is induced in cancer cells by β1,3-*N*-acetylglucosaminyltransferase 6 (β3Gn-T6), which is an essential enzyme for the synthesis of core 3 *O*-glycan [8], has been reported to reduce malignant characteristics (proliferation, invasion, and metastasis) in colon, prostate, and pancreatic cancers according to *in vitro* assays with animal model experiments [8, 21, 22]. However, clinicopathological significance of core 3 *O*-glycan and β3Gn-T6 has not been evaluated yet.

The aim of this study was to investigate the clinicopathological impact of core 3 *O*-glycan on PDAC through immunohistochemical detection of β3Gn-T6, rather than measuring the structure of core 3 *O*-glycan, for which currently there is no specific antibody or lectin. We examined the expression of β3Gn-T6 in 156 consecutive cases of PDAC along with normal pancreatic tissue and the most popular premalignant lesion of pancreatic intraepithelial neoplasia (PanIN) [23], and compared the clinicopathological features. We also examined the clinicopathological impact of several *O*-glycans in PDAC.

## Materials and methods

### Ethics approval and consent to participate

This study was approved by the Institutional Review Board of the National Cancer Center, Japan (#2005–077). The written informed consent was obtained from all participants involved in the study, and all clinical investigations were conducted in line with the principles of the Declaration of Helsinki.

### Study population

Clinical and pathological data and specimens used for this study were obtained through a detailed retrospective review of the medical records of 156 consecutive patients with PDAC who had undergone surgical resection between 2009 and 2011 at the National Cancer Center Hospital, Tokyo. None of the patients had received any therapy before surgery. All patients included in this study underwent macroscopic curative resection, and all cases involved conventional ductal carcinomas. The clinicopathological characteristics of the study participants are summarized in Table 1. The median follow-up period after surgical treatment was 29.1 (1.7–126.5) months. Recurrence was suspected when a new local or distant metastatic lesion was found on serial images, and an increase in tumor marker levels was observed. At the census date (September 2018), we checked whether the patients were dead or alive; 62 patients (39.7%) were alive, 82 (52.6%) had died of pancreatic cancer, and 12 (7.6%) had died of other causes. All M1 (TNM classification [24]) patients showed only nodal metastasis around the abdominal aorta.

**Table 1:** Correlations between Exp-scores of β3Gn-T6 and clinicopathological variables.

Variable		Number (%)	Median Exp-score of β3Gn-T6	*P* value
Gender	Male	90 (57.7%)	30.0	0.32^a^
	Female	66 (42.3%)	45.0	
Age (years, median 68.5)	70–89	78 (50.0%)	40.0	0.60^a^
	38–69	78 (50.0%)	30.0	
Adjuvant chemotherapy	Presence	111 (71.2%)	30.0	0.42^b^
	Absence	44 (28.2%)	47.5	
	Unknown	1 (0.6%)	85	
Tumor size (mm, median 35)	35–96	84 (53.8%)	40.0	0.33^a^
	13–34	72 (46.2%)	30.0	
Tumor grade	G1	44 (28.2%)	55.0	<0.0005^b^
	G2	85 (54.5%)	40.0	
	G3	27 (17.3%)	10.0	
Lymphatic invasion	ly2, ly3	131 (84.0%)	37.5	0.58^a^
	ly0, ly1	25 (16.0%)	35.0	
Venous invasion	v2, v3	117 (75%)	35.0	0.79^a^
	v0, v1	39 (25%)	35.0	
Intrapancreatic neural invasion	ne2, ne3	97 (62.2%)	35.0	0.13^a^
	ne0, ne1	59 (37.8%)	32.5	
Nerve plexus invasion	Presence	54 (34.6%)	45.0	0.086^a^
	Absence	102 (65.4%)	35.0	
Lymph node metastasis	N1, N2	116 (74.4%)	35.0	0.92^a^
	N0	40 (25.6%)	47.5	
Disatant metastasis	M1	9 (5.8%)	25.0	0.78^a^
	M0	147 (94.2%)	35.0	
Surgical margin	Positive	45 (28.8%)	40.0	0.55^a^
	Negative	111 (71.2%)	35.0	

^a^Mann-Whitney-U test

^b^Kruskal-Wallis test

### Pathological evaluation

All carcinomas were examined pathologically and classified according to the World Health Organization (WHO) classification [11, 23], Union for International Cancer Control (UICC) TNM classification [24], and the Classification of Pancreatic Carcinoma of the Japan Pancreas Society [25]. Surgically resected specimens were fixed in 10% formalin and cut into serial 5-mm-thick slices and all sections were stained with hematoxylin and eosin (HE) for pathological examination. Representative tissue blocks were selected for subsequent analyses. We used PanINs and normal pancreatic tissue in this study as follows: the areas containing PanIN were apart from cancer cells during microscopic observation, and normal pancreatic tissues were more than 2 cm away from the tumor cells.

### Immunohistochemistry and lectin-histochemistry

Immunohistochemistry was performed on 4-μm-thick formalin-fixed paraffin-embedded tissue sections using the avidin–biotin complex method as described previously [26]. Lectin-histochemical analysis was performed in the same way as the immunohistochemical analysis, except lectin was used instead of the primary antibody. The primary antibodies and lectins used in this study are listed in Table 2. Immunohistochemical analysis without the primary antibody was carried out as a negative control. Positive findings are shown in S1 Fig.

**Table 2:** Primary antibodies and lectins.

antigen	antibody clone, lectin	type	dilution	internal positive control	source
β3Gn-T6	G8-144	Mouse IgG	1:2000	goblet cells of duodenal epithelium	in house (8)*
Tn antigen	NCC-LU-35-65	Mouse IgM	1:100	surface epithelial cells of duodenal mucosa	in house (32)*
6-sulfo *N*-acetyllactosamine on extended core 1 *O*-glycan	MECA-79	Rat IgM	1:100	endothelial cells of high endothelial venule of lymph node	BD Biosciences
sLeX	CSLEX1	Mouse IgM	1:1000	Brunner’s glands of duodenum	BD Biosciences
sLeX	HECA-452	Rat IgM	1:5	Brunner’s glands of duodenum	ATCC
sLeX	NCC-ST-439	Mouse IgM	1:5	Brunner’s glands of duodenum	in house (19)*
MUC5AC	CLH2	Mouse IgG	1:100	Fovolar cells of gastric mucosa	Leica
T antigen	*Peanut aggulutinin*	lectin	1:100	Brunner’s glands of duodenum	VECTOR
non-reducing terminus of *N*-acetylglucosamine	*Griffonia simplicifolia—II*	lectin			VECTOR

β3Gn-T6: β1,3-*N*-acetylglucosaminyltransferase 6, sLeX: sialyl Lewis X antigen

ATCC: American type culture collection, Leica: Leica Biosystems, VECTOR: Vector laboratories

*() reference number

### Evaluation of immunohistochemistry and lectin-histochemistry

After the immunohistochemical and lectin-histochemical analyses, the antigen expression levels were assessed via a semiquantitative scoring system that incorporated percentages of stained cells with the categorized staining intensity. The staining intensity was recorded in comparison to internal positive controls as 0, negative; 1+, positive but weaker than an internal positive control; 2+, equal to the internal positive control; and 3+, stronger than the internal positive control. The percentage of stained cells was determined by the comparison of the number of cancer cells with each staining intensity to the total number of cancer cells. The sum of products obtained by multiplying the staining intensity and the percentage of corresponding intensity was defined as an expression score (Exp-score). Two observers, i.e., Japanese certified pathologists (ND and NH), who had no access to the patient data, independently evaluated the Exp-score. For statistical analyses, patients were subdivided into two groups by means of the medians as a cutoff.

### Cell culture

Human pancreatic cancer cell lines, Capan-1, Capan-2, CFPAC-1, and AsPC-1, were obtained from the American Type Culture Collection (ATCC). Capan-1, Capan-2, and CFPAC-1 cells were cultured in Dulbecco’s modified Eagle’s medium (DMEM) supplemented with 10% of fetal calf serum at 37°C and 5% CO_2_ in a humidified atmosphere. AsPC-1 cells were cultured under the same conditions except the RPMI 1640 medium instead of DMEM medium.

### Gene transduction

To generate lenti-viral expression vectors, a segment encoding 3'ΔLTR (between Kpn I and Stu I) of pCDH-MCS-T2A-copGFP-MSCV (SBI system biosciences, Palo Alto, CA) was exchanged by segment encoding 3'ΔLTR (Kpn I and Stu I fragment) of pLenti7.3 (Invitrogen); then, the Cla I and Nhe I segment encoding the CMV promoter, amplified by PCR using pLenti7.3 as a template, was inserted between the 5'LTR encoding and multi-cloning sites, resulting in pCDH-CMV-MCS-T2A-copGFP. The EcoR I and Nco I segment encoding the internal ribosome entry site (IRES) sequence from pIRES-hrGFP2a (Clontech) was subcloned into pcDNA3.1/Zeo(+) (Invitrogen), resulting in pcDNA3.1/IRES-Zeo. The EcoR I and Not I segment containing the IRES and Zeo resistance selection cassette from pcDNA3.1/IRES-Zeo was subcloned into pCDH-CMV-MCS-T2A-copGFP and resulted in pCDH-CMV-MCS-IRES-Zeo-T2A-copGFP. The Nhe I and Xho I fragment from pcDNA3.1-B3GnT6, *B3GNT6* (encoding β3Gn-T6) expression vector [8], was subcloned into pCDH-CMV-MCS-IRES-Zeo-T2A-copGFP and resulted in pCDH-CMV-MCS-IRES-Zeo-T2A-copGFP/huB3GnT6. 293FT cells (Invitrogen) were co-transfected with psPAX2, pMD2.G, and pCDH-CMV-MCS-IRES-Zeo-T2A-copGFP/huB3GnT6 or pCDH-CMV-MCS-IRES-Zeo-T2A-copGFP using Lipofectamine LTX with Plus reagent (Invitrogen) according to the manufacturer’s instruction. Seventy-two hours after transfection, the culture supernatants were harvested and concentrated using Lenti-X Concentrator (Takara Bio, Kusatsu, Japan) as the virus solution. For viral infection and gene transduction, Capan-1 cells were seeded into 6-well culture plates at a density of 3.0 × 10^5^ cells per well and cultured with the prepared viral solution (1:50) with polybrene (2 to 8 μg/mL) at 37°C for 24 hours. Viral-transduced cells were selected using Zeocin (Invitrogen) at 200 μg/mL for 10 days. Stably *B3GNT6* and mock transduced Capan-1 cells were named as Capan1-B3GnT6 and Capan1-mock, respectively.

### Immunofluorescence

Cells were seeded on a chamber slide. The staining procedure was previously described [27]. Immunofluorescence images were obtained using a BZ-X710 all-in-one fluorescence microscope (Keyence, Japan).

### Extraction of RNA and quantitative RT-PCR (qRT-PCR)

Total RNA was extracted from pancreatic cancer cells, as described previously [28]. All samples were treated with rDNase during isolation, in accordance with the manufacturer’s instructions. qRT-PCR for target genes and non-target housekeeping control genes was performed with a Quantstudio 3 (Thermo scientific) using FastStart Universal Probe Master (ROX) and probes from the Universal Probe Library (Roche Diagnostics Corp., Indianapolis, IN), as described previously [26]. The sequences of the primers and the respective Universal Probe Library probes are given in S1 Table. The CT values were normalized to that of GAPDH, and the ΔΔCT method was utilized to compare the expression levels of the genes.

### Immunoprecipitation and western blot analysis

Whole-cell lysates of Capan1-B3GnT6 and Capan1-mock cells were immunoprecipitated with an anti-MUC5AC antibody (45M1, Abcam, Cambridge, UK) according to the literature [29]. To analyze the structures of glycans attached to MUC5AC, immune complexes were subjected to western blot analysis. The immune complexes were separated by SDS-PAGE in a 4–12% gradient gel (Invitrogen) and were transferred to a Polyvinylidene fluoride (PVDF) membrane, which was then blocked by incubation with PBS-Tween containing 5% of bovine serum albumin as described elsewhere [30]. After that, the membrane was incubated with a primary antibody and biotin-conjugated secondary antibody or with biotin-conjugated lectins (Table 2) followed by ABC reagents (Vector laboratories).

### Statistical analysis

Comparison analyses were performed using the nonparametric test. Post-operative overall survival (OS) and disease-free survival (DFS) rates were calculated using the Kaplan-Meier method and analyzed by the log-rank test. The factors found to be significant by univariate analysis were subjected to multivariate analysis using the Cox proportional hazards model (backward elimination method). Differences at *P*<0.05 were considered statistically significant. Statistical analyses were performed using SPSS software version 26 (IBM Corp., Armonk, USA).

## Results

### Expression of glycans and β3Gn-T6 in PDAC cells, premalignant cells, and normal tissues

**Fig 2:**
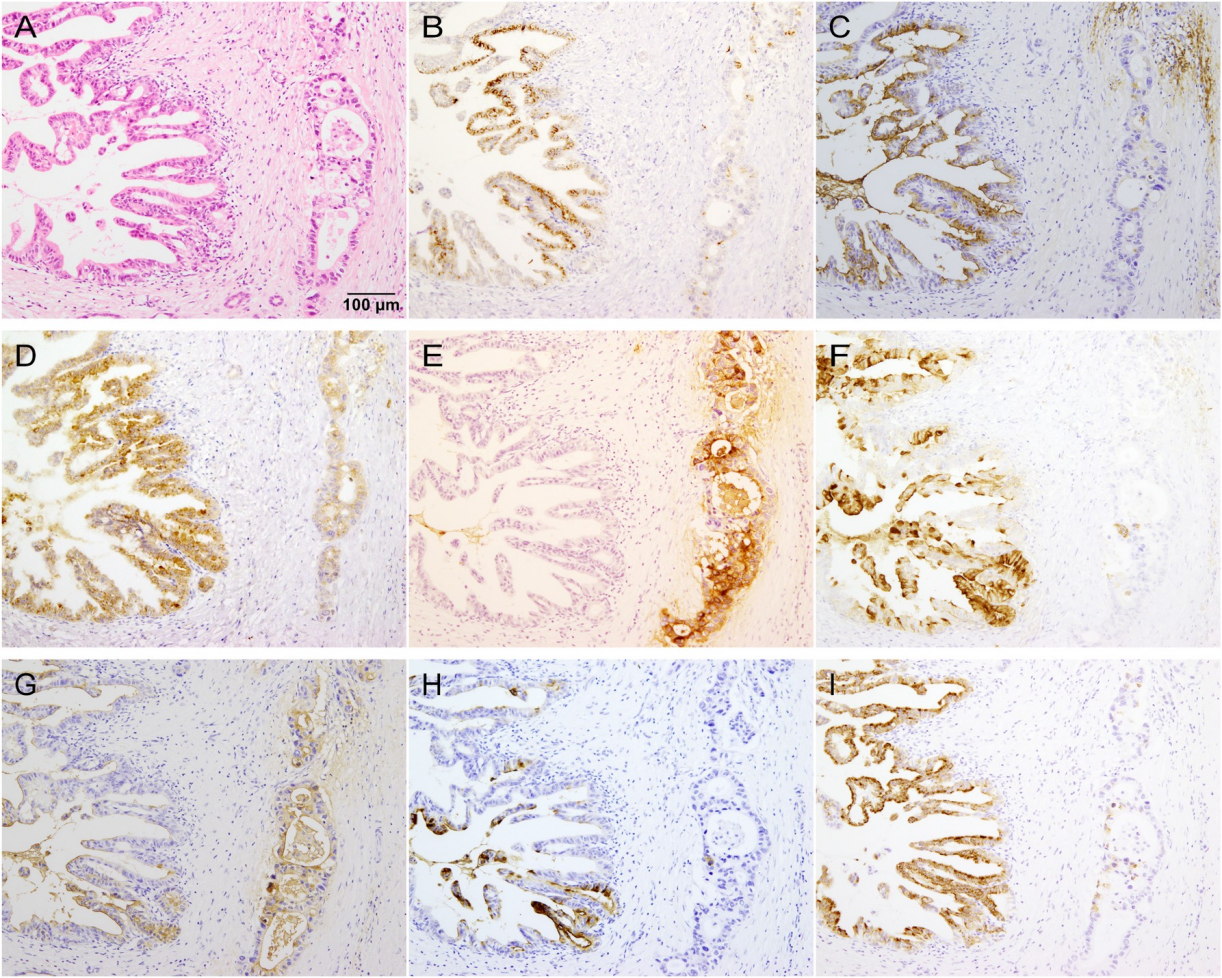
Representative microscopic images of immunohistochemistry and lectin-histochemistry in PDAC tissues. Middle-power view of tissues stained with (A) HE, (B) β3Gn-T6, (C) T antigen (PNA), (D) Tn antigen (LU-35), (E) 6-sulfo *N*-acetyllactosamine on extended core 1 *O*-glycan (MECA-79), (F) sLeX (CSLEX1), (G) sLeX (HECA-452), (H) sLeX on core 2 *O*-glycan (ST-439) and (I) MUC5AC. All these sections show the same region of PDAC tissue. In the left half of the panel, intraductal spreading of adenocarcinoma cells is seen, and invasive adenocarcinomas are present in the right half. All these antigens are expressed to various degrees of heterogeneity in the adenocarcinoma cells, even within the same case. Different staining patterns of sLeX are observed depending on the specificity of the antibodies used as described in the main text (F–H).

**Fig 3:**
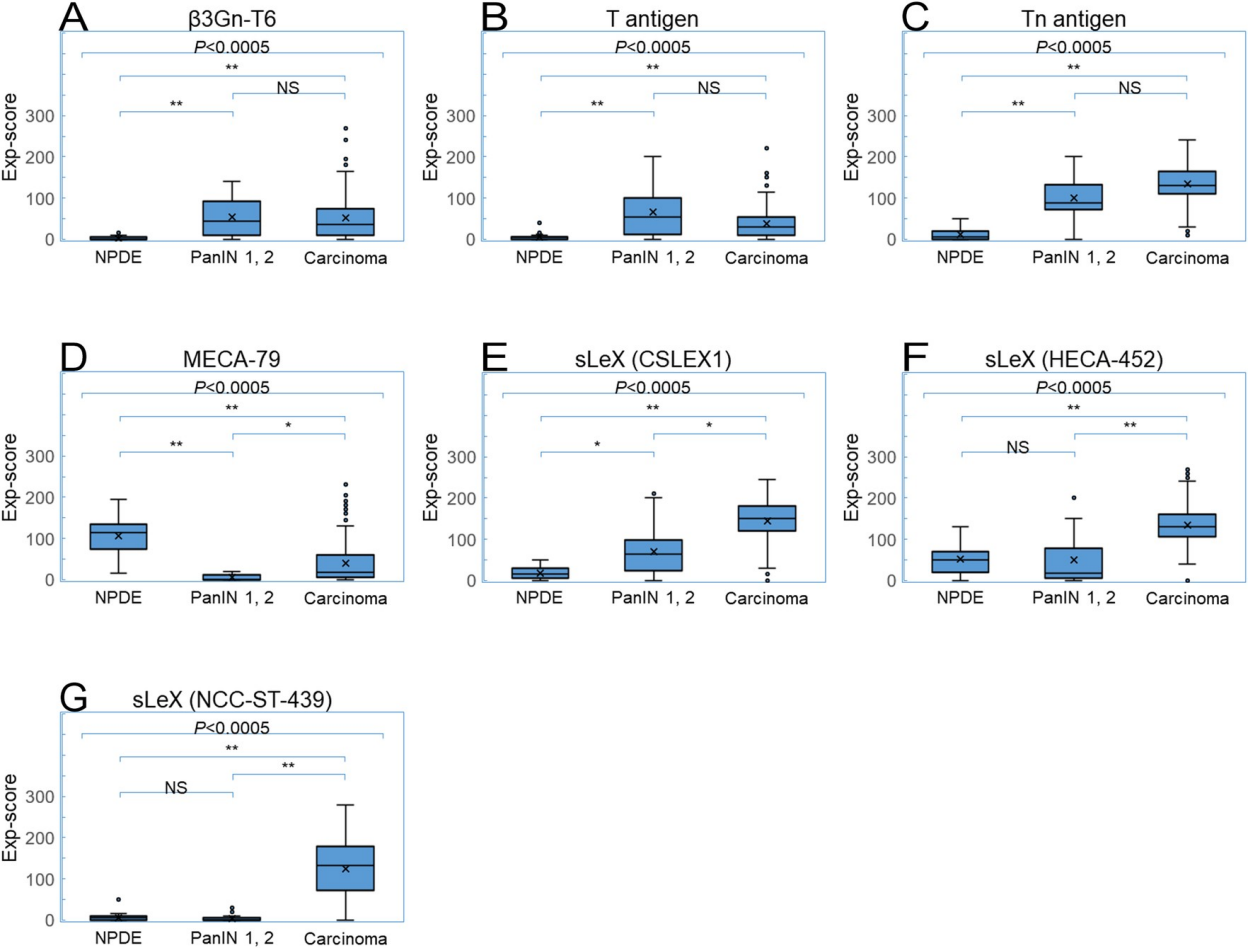
Comparison of glycan antigens and β3Gn-T6 expression among PDACs (n = 156), PanINs (n = 26), and normal pancreatic tissues (n = 35). ((A) β3Gn-T6, (B) T antigen (PNA), (C) Tn antigen (LU-35), (D) 6-sulfo *N*-acetyllactosamine on extended core 1 *O*-glycan (MECA-79), (E) sLeX (CSLEX1), (F) sLeX (HECA-452), and (G) sLeX on core 2 *O*-glycan (ST-439). Boxes represent medians and interquartile ranges. Crosses represent mean values. Whiskers represent the minimum and maximum 1.5 interquartile ranges. Circles represent extremes. All glycan antigens, except MECA-79 antigen, are expressed significantly more highly in PDAC cells than in NPDEs. MECA-79 antigen expression in PDAC cells is significantly lower than that in NPDEs. Exp-scores were compared and analyzed using the Friedman’s test.

We evaluated the expression of the T antigen (staining with Peanut agglutinin, PNA) [31], Tn antigen [staining with antibody NCC-LU-35 (LU-35)] [32], 6-sulfo *N*-acetyllactosamine on extended core 1 *O*-glycan (staining with antibody MECA-79) [3, 17], sLeX (staining with antibodies CSLEX1 and HECA-452) [33], sLeX on core 2 *O*-glycan (staining with antibody ST-439) [18, 19], and β3Gn-T6 (Fig 1) in PDAC cells, PanINs, and noncancerous tissues, normal pancreatic duct epithelial cells (NPDEs), and other normal tissues. Representative immunohistochemical and lectin-histochemical features are shown in Fig 2 and S1 Fig. PDAC is usually composed of variously differentiated cancer cells, with varied frequency and intensity of glycan expression in PDAC cells in the same case. We first analyzed glycan expression in each component of PDAC (Table 3). Next, to determine a representative value for overall expression of antigens in PDAC cells in each PDAC case, we calculated the Exp-score. All glycan antigens, except MECA-79 antigen, were expressed significantly more highly in PDAC cells than in NPDEs (Fig 3). In contrast, MECA-79 antigen expression in PDAC cells was significantly lower than that in NPDEs (Fig 3).

**Table 3:** Summary of glycan and its related antigen expression.

antigens		β3Gn-T6	T antigen	Tn antigen	MECA-79*	sLeX			MUC5AC
						(CSLEX1)	(HECA-452)	(ST-439)	
PDAC components									
	Well differentiated adenocarcinoma	10/38 (26.3%)	3/38 (7.9%)	29/38 (76.3%)	1/38 (2.6%)	25/38 (65.8%)	32/38 (84.2%)	14/38 (36.8%)	17/38 (44.7%)
	Moderately differentiated adenocarcinoma	7/38 (18.4%)	4/38 (10.5%)	35/38 (92.1%)	3/38 (7.9%)	35/38 (92.1%)	37/38 (97.3%)	30/38 (78.9%)	6/38 (15.8%)
	Poorly differentiated adenocarcinoma	0/26 (0%)	6/26 (23.1%)	17/26 (65.4%)	2/26 (7.7%)	20/26 (76.9%)	21/26 (80.8%)	17/26 (65.4%)	1/26 (3.8%)
	Squamous cell carcinoma	0/6 (0%)	0/6 (0%)	1/6 (16.7%)	0/6 (0%)	0/6 (0%)	0/6 (0%)	0/6 (0%)	0/6 (0%)
Premalignant lesion									
	PanIN 1	7/21 (33.3%)	10/21 (47.6%)	17/21 (81.0%)	0/21 (0%)	8/21 (38.1%)	5/21 (23.8%)	0/21 (0%)	7/21 (33.3%)
	PanIN 2	7/17 (41.2%)	5/17 (29.4%)	12/17 (70.6%)	0/17 (0%)	4/17 (23.5%)	6/17 (35.3%)	0/17 (0%)	8/17 (47.1%)
Normal tissue									
	Pancreatic duct epithelial cell	0/35 (0%)	0/35 (0%)	1/35 (2.9%)	31/35 (88.6%)	0/35 (0%)	10/35 (28.6%)	1/35 (2.9%)	0/35 (0%)
	Acinar cell	0/35 (0%)	4/35 (11.4%)	14/35 (40.0%)	0/35 (0%)	1/35 (2.9%)	10/35 (28.6%)	0/35 (0%)	0/35 (0%)
	Islet cell	0/36 (0%)	9/36 (25.0%)	1/36 (2.8%)	0/36 (0%)	2/36 (5.6%)	0/36 (0%)	1/36 (2.8%)	0/36 (0%)

Cancer tissues of 40 cases selected randomly are subdivided into each component. Premalignant lesions were selected in non-cancerous area of the pancreas in PDAC cases. Normal pancreatic tissue at least 2 cm far from cancer cells are used in this assay. The number of tissues in which more than 50% of each component is positive for each antigen out of total tissues which contain each component is indicated.

*6-sulfo *N*-acetyllactosamine on extended core 1 O-glycan

The T antigen: This antigen was found to be expressed in some of the well or moderately differentiated PDAC cells but not in NPDEs. Over 30% of low-grade PanINs expressed the T antigen (Table 3). T antigen expression was mildly higher in PDAC cells compared to NPDEs (Fig 3).

The Tn antigen: The majority of PDAC cells and PanINs expressed the Tn antigen, but most NPDEs did not (Table 3). Tn antigen expression in PDAC cells was markedly higher, and most of the cases were strongly positive, i.e., the Exp-score was >100 (Fig 3).

MECA-79 antigen: In contrast to NPDEs, which usually express MECA-79 antigen, PDAC cells were found to rarely express it, whereas PanINs did not express it at all (Table 3 and Fig 3).

SLeX: PDAC cells expressed sLeX strongly and at a high frequency, regardless of the antibodies applied. However, PanINs and NPDEs showed different profiles depending on the antibodies used (Table 3 and Fig 3). Antibody ST-439 identified the limited sLeX antigen, sLeX on core 2 *O*-glycan, so that ST-439^+^ PDAC cells also stained with antibodies CSLEX1 or HECA-452. Antibodies CSLEX1 and HECA-452 recognize both *O*-linked and *N*-linked sLeX. Antibodies HECA-452 and ST-439, but not CSLEX1, can recognize sulfated sLeX [2, 18], These features can be summarized: (1) both staining frequency and area were ranked as follows, in ascending order: HECA-452, CSLEX1, and ST-439; (2) potentially sulfated sLeX was found in MECA-79^+^ NPDEs, where HECA-452 staining was sometimes present while CSLEX1 staining was not; (3) low-grade PanINs were positive for HECA-452 staining and CSLEX1 staining, and normal epithelial cells were positive for HECA-452 staining but almost negative for ST-439 staining. These results suggest that PanINs and epithelial cells did not express core 2 *O*-glycan.

**Fig 4:**
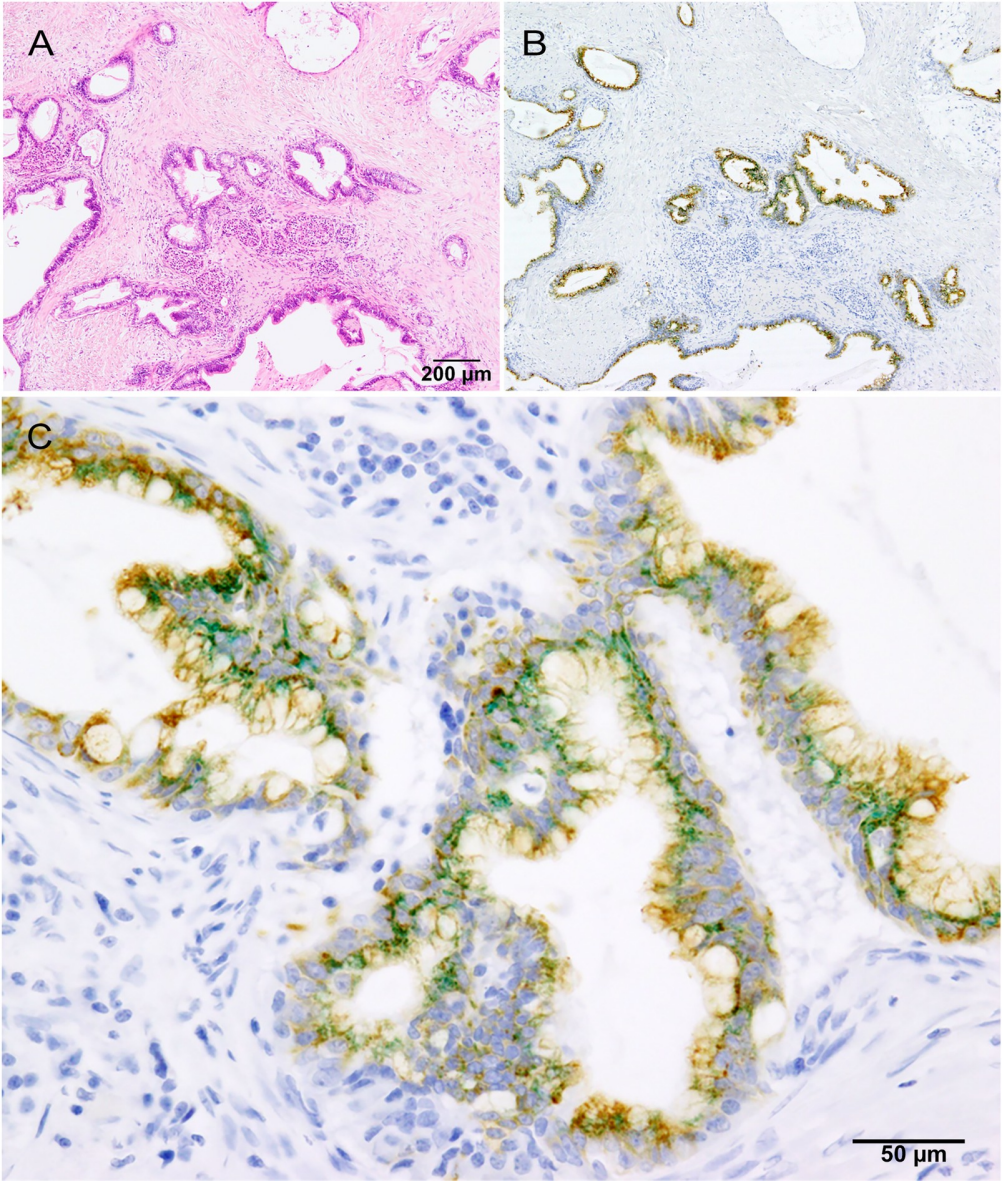
Double immunohistochemistry for β3Gn-T6 and MUC5AC. Low-power view of histological staining (A) and double immunohistochemical staining (B) and high-power view of double immunohistochemical staining (C). Dotlike staining of β3Gn-T6 (green) and membranous staining of MUC5AC (brown) are often present in the same adenocarcinoma cells.

β3Gn-T6: β3Gn-T6 was expressed in ~20% of PDAC cells and 30–40% of low-grade PanINs but not in NPDEs (Table 3). Higher histological differentiation was associated with a higher level of β3Gn-T6^+^ in PDAC cells. NPDEs typically have a pancreatobiliary phenotype, whereas low-grade PanINs usually have a gastric phenotype [23, 34]. Furthermore, β3Gn-T6 is normally expressed in normal gastric foveolar cells as well as colonic goblet cells [8]. It is possible that β3Gn-T6 is expressed in cells with the gastric foveolar phenotype. When double immunohistochemical staining for β3Gn-T6 and MUC5AC was performed, both antigens were often found to be expressed in the same PDAC cells (Fig 4). In addition, there was a significant correlation between them, with a high correlation coefficient (ρ = 0.49; Table 4).

**Table 4:** Correlations among glycans and related proteins (Spearman's correlation coefficient value).

	β3Gn-T6	T antigen	Tn antigen	MECA-79***	sLeX		
					(CSLEX1)	(HECA-452)	(ST-439)
T antigen	R = 0.16^*^						
Tn antigen	R = 0.066	R = 0.13					
MECA-79***	R = -0.17^*^	R = 0.13	R = -0.032				
sLeX (CSLEX1)	R = 0.064	R = -0.047	R = 0.081	R = 0.11			
sLeX (HECA-452)	R = -0.11	R = -0.075	R = 0.034	R = -0.080	R = 0.38^**^		
sLeX (ST-439)	R = 0.036	R = -0.0010	R = 0.13	R = 0.13	R = 0.69^**^	R = 0.32^**^	
MUC5AC	R = 0.49^**^	R = 0.19^*^	R = 0.19^*^	R = -0.20^*^	R = 0.12	R = 0.016	R = 0.15

*Correlation is significant at the 0.05 level (2-tailed)

**Correlation is significant at the 0.01 level (2-tailed).

***6-sulfo *N*-acetyllactosamine on extended core 1 O-glycan

### Relation between glycan expression and clinicopathological variables

The correlation between glycan expression in PDAC cells and various clinicopathological factors was examined next. Significant correlations were found only between higher β3Gn-T6 expression in PDAC cells and a lower histological grade, between higher MECA-79 antigen expression and a higher histological grade, and between higher sLeX expression (staining with antibody HECA-452) and a lower histological grade (Table 1 and S2 Fig).

Next, we evaluated correlations among expression levels of different glycans. We compared the Exp-scores of all glycans by Spearman’s test (Table 4). Scores on sLeX detected by different antibodies correlated positively. In addition, a few significant correlations were found between β3Gn-T6 and MUC5AC (ρ = 0.49) and between β3Gn-T6 and T antigen (ρ = 0.16), and a negative correlation was found between β3Gn-T6 and MECA-79 antigen (ρ = −0.17).

### MUC5AC carries core 3 *O*-glycan generated by *B3GNT6* gene expression in pancreatic cancer cells

**Fig 5:**
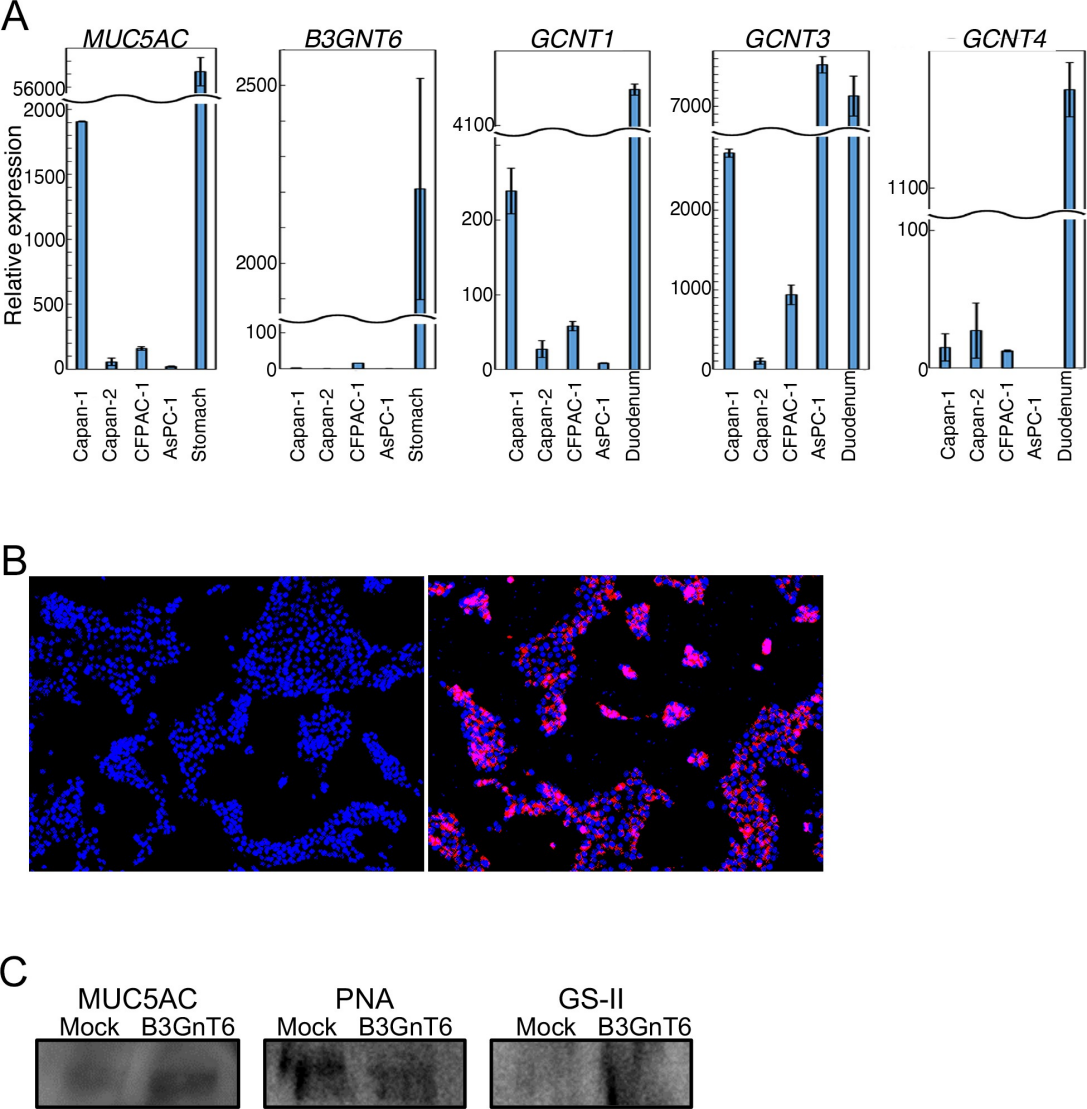
MUC5AC has core 3 *O*-glycan generated by *B3GNT6* gene expression in pancreatic cancer cells. (A) Comparison of expression of genes *MUC5AC*, *B3GNT6*, and core 2 *O*-glycan synthase among PDAC cells. Capan-1 cells express *MUC5AC* more highly than the other cell lines but do not express *B3GNT6*. (B) Capan1-B3GnT6 cells (right panel) but not Capan1-mock cells (left panel) express β3Gn-T6 as detected by the immunofluorescence assay (red). Nuclei are stained by 4’,6-diamindino-2-phenylindole (DAPI, blue). (C) MUC5AC immunoprecipitated with the anti-MUC5AC antibody was subjected to SDS-PAGE and transferred to a nitrocellulose membrane, then the membrane was blotted with GS-II, PNA, or the anti-MUC5AC antibody. The level of nonreducing terminal GlcNAc is higher in Capan1-B3GnT6 cells compared to Capan1-mock cells, even though the MUC5AC amount is comparable between these two cell lines. Conversely, the T antigen level is lower in Capan1-B3GnT6 cells compared to Capan1-mock cells.

To investigate whether MUC5AC contains core 3 *O*-glycan, we examined glycosylation status of MUC5AC. To select suitable pancreatic cancer cells for the assay, we analyzed the expression of genes *B3GNT6* (encoding β3Gn-T6) and *MUC5AC*, together with genes encoding core 2 synthases (*GCNT1*, *GCNT3*, and *GCNT4)* by qRT-PCR. We chose Capan-1 cells because they show almost no expression of *B3GNT6* and higher expression of *MUC5AC* compared to the other cell lines (Fig 5A). The immunofluorescence assay revealed that GFP-positive stably *B3GNT6*-transduced cells, Capan1-B3GnT6 expressed the β3Gn-T6 protein (Fig 5B). The *N*-acetylglucosaminyl terminus of core 3 *O*-glycan can be detected by a lectin called GS-II [21]. MUC5AC that was immunoprecipitated from the lysates of Capan1-B3GnT6 and Capan1-mock cells was subjected to SDS-PAGE followed by western blotting with GS-II (Fig 5C). An intense band was produced by MUC5AC isolated from Capan1-B3GnT6 cells. In contrast, no band was yielded by the MUC5AC isolated from Capan1-mock cells, even though anti-MUC5AC stained bands were comparable between them. These results indicated that MUC5AC from Capan1-B3GnT6 had core 3 *O*-glycan. In support of this finding, PNA binding to MUC5AC was lower in Capan1-B3GnT6 cells compared with Capan1-mock cells (Fig 5C). It was confirmed that core 3 *O*-glycan was present on MUC5AC isolated from Capan1-B3GnT6 cells, and that this glycan was synthesized by β3Gn-T6.

### Prognostic significance of glycan-related antigens in PDAC cells

**Fig 6:**
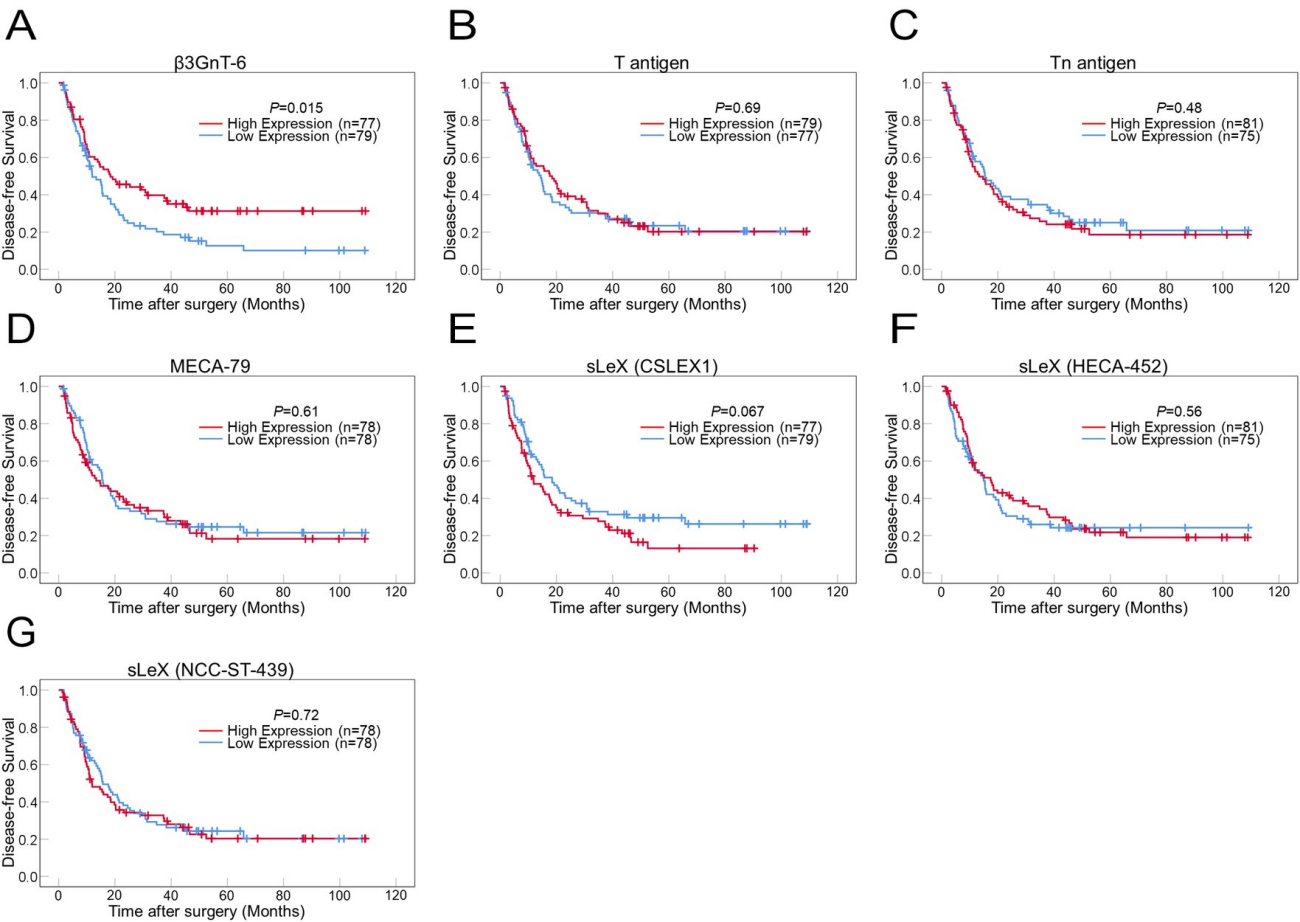
Kaplan-Meier survival curves for disease-free survival in patients with PDAC according to (A) β3Gn-T6, (B) T antigen (PNA), (C) Tn antigen (LU-35), (D) 6-sulfo *N*-acetyllactosamine on extended core 1 *O*-glycan (MECA-79), (E) sLeX (CSLEX1), (F) sLeX (HECA-452), and (G) sLeX on core 2 *O*-glycan (ST-439). Patients having PDAC with higher expression of β3Gn-T6 (red line) show a significantly longer survival compared to those with lower expression of β3Gn-T6 (blue line) in A. Patients having PDAC with higher expression of sLeX (CSLEX1) (red line) show a tendency to be shorter survival compared to those with lower expression of sLeX (CSLEX1) (blue line) in E. The other antigens are not significantly associated with patient outcome.

Kaplan–Meier survival analyses revealed a statistically significant association between higher expression of β3Gn-T6 in PDAC cells and longer DFS (Fig 6). Patients with higher sLeX (staining with CSLEX1) expression tended to have shorter DFS. No significant association was found between any other glycan expression and patient outcomes (DFS or OS). No significant association was found between OS and β3Gn-T6 expression (S3 Fig).

Cox proportional analysis of the groups categorized by each glycan expression and β3Gn-T6 expression in PDAC cells as well as conventional clinicopathological variables are shown in Table 5. Data on variables found to be significant by univariate analysis were subjected to multivariate analysis. In the latter, several variables (age, lymph node metastasis, nerve plexus invasion, tumor histological grade, and β3Gn-T6 expression) were found to be significantly associated with DFS.

**Table 5:** Univariate and multivariate analyses of prognostic factors associated with disease-free survival (A) and and overall survival (B) in patients with pancreatic ductal adenocarcinoma (n = 156).

	Cox univariate analysis			Cox multivariate analysis	
	HR (95%CI)	*P* value		HR (95%CI)	*P* value
Gender: Male vs. Female	0.9 (0.62–1.3)	0.6			
Age (year): ≤69 vs. ≥70	0.56 (0.39–0.81)	**0.0024**	** **	0.66 (0.45–0.97)	**0.036**
Adjuvant chemotherapy: No vs. Yes	0.82 (0.54–1.2)	0.34			
Tumor size (mm): <35 vs. ≥35	1.5 (1.0–2.1)	**0.042**	** **		
Tumor grade: 1 vs. 2 and 3	1.8 (1.2–2.9)	**0.0068**	** **	1.8 (1.2–2.9)	**0.0069**
Lymphatic invasion: Low vs. High	1.3 (0.74–2.2)	0.4			
Venous invasion: Low vs. High	1.7 (1.1–2.7)	**0.02**	** **		
Intrapancreatic neural invasion: Low vs. High	1.6 (1.1–2.3)	**0.028**	** **		
Nerve plexus invasion: Low vs. High	1.7 (1.1–2.4)	**0.0085**	** **	1.6 (1.1–2.4)	**0.022**
Lymph node metastasis: No vs. Yes	2.2 (1.4–3.6)	**0.0012**	** **	1.7 (1.0–2.9)	**0.035**
Distant metastasis: No vs. Yes	1.6 (0.74–3.4)	0.24			
Surgical margin: Negative vs. Positive	1.4 (0.97–2.2)	0.07			
β3Gn-T6: Low vs. High	0.63 (0.43–0.92)	**0.016**	** **	0.65 (0.44–0.95)	**0.024**
T antigen: Low vs. High	0.93 (0.64–1.3)	0.69			
Tn antigen: Low vs. High	1.1 (0.79–1.7)	0.48			
MECA-79*: Low vs. High	1.1 (0.76–1.6)	0.61			
sLeX (CSLEX1): Low vs. High	1.4 (0.97–2.0)	0.068			
sLeX (HECA-452): Low vs. High	0.9 (0.62–1.3)	0.56			
sLeX (ST-439): Low vs. High	1.1 (0.74–1.5)	0.72			
(B)					
	Cox univariate analysis			Cox multivariate analysis	
	HR (95%CI)	*P* value		HR (95%CI)	*P* value
Gender: Male vs. Female	0.84 (0.53–1.3)	0.45			
Age (year): ≤69 vs. ≥70	0.61 (0.38–0.97)	**0.037**	** **	0.6 (0.37–0.96)	**0.035**
Adjuvant chemotherapy: No vs. Yes	0.76 (0.45–1.3)	0.3			
Tumor size (mm): <35 vs. ≥35	2.2 (1.3–3.5)	**0.0016**	** **	2.0 (1.2–3.2)	**0.0067**
Tumor grade: 1 vs. 2 and 3	2.2 (1.3–4.0)	**0.0053**	** **	2.3 (1.3–4.0)	**0.0054**
Lymphatic invasion: Low vs. High	1.1 (0.58–2.1)	0.78			
Venous invasion: Low vs. High	1.8 (1.0–3.2)	**0.037**	** **		
Intrapancreatic neural invasion: Low vs. High	1.5 (0.96–2.5)	0.075			
Nerve plexus invasion: Low vs. High	1.9 (1.2–3.1)	**0.0054**	** **	1.7 (1.1–2.8)	**0.022**
Lymph node metastasis: No vs. Yes	1.9 (1.0–3.3)	**0.037**	** **		
Distant metastasis: No vs. Yes	1.2 (0.48–3.0)	0.69			
Surgical margin: Negative vs. Positive	1.7 (1.1–2.8)	**0.027**	** **		
β3Gn-T6: Low vs. High	0.82 (0.52–1.3)	0.4			
T antigen: Low vs. High	0.85 (0.54–1.3)	0.48			
Tn antigen: Low vs. High	1.4 (0.87–2.2)	0.17			
MECA-79*: Low vs. High	0.97 (0.61–1.5)	0.89			
sLeX (CSLEX1): Low vs. High	1.3 (0.8–2.0)	0.32			
sLeX (HECA-452): Low vs. High	0.74 (0.47–1.2)	0.2			
sLeX (ST-439): Low vs. High	1.1 (0.71–1.8)	0.64			

*6-sulfo *N*-acetyllactosamine on extended core 1 O-glycan

## Discussion

Mucin-type *O*-glycan is known to be involved in tumor development and malignant characteristics. However, its clinicopathological significance has not yet been sufficiently elucidated. Here, we investigated the clinicopathological significance of both *O*-glycan cores and peripheral modified glycans in PDAC. Higher β3Gn-T6 expression was noted in more differentiated adenocarcinoma in PDAC patients. These PDAC cases showed significantly longer DFS. Together with previous reports indicating that forced expression of β3Gn-T6 reduces the aggressiveness of cancers *in vitro* and *in vivo* [8, 21, 22], our findings suggest that β3Gn-T6 expression in PDAC cells is a favorable prognostic indicator. In addition, the expression of β3Gn-T6 in PDAC cells and PanINs significantly correlated with the expression of MUC5AC in these cells, implying that β3Gn-T6 expression is related to cellular differentiation status of the gastric foveolar phenotype. The expression of the T antigen, Tn antigen, sLeX antigen, and sLeX on core 2 *O*-glycan was higher in PDAC cells. Unexpectedly, we did not find any significant association with patient outcome in our cohort. However, 6-sulfo *N*-acetyllactosamine on extended core 1 *O*-glycan (MECA-79 antigen) was underexpressed in PDAC cells compared to NPDEs and was not associated with patient outcome.

Core 3 *O*-glycan is widely distributed throughout the gastrointestinal tract [35] and is synthesized only by β3Gn-T6 expressed normally in gastric foveolar epithelial cells and colonic goblet cells [8]. β3Gn-T6 is not expressed in normal pancreatic tissue, and the induction of β3Gn-T6 has been previously found in some low-grade PanINs and differentiated PDAC cells (Table 3). Takano et al. have reported that MUC5AC^+^ PDAC tends to be a more differentiated adenocarcinoma [36]. In our study, these tumors were found to start expressing β3Gn-T6, which significantly correlated with MUC5AC expression (Fig 4 and Table 4). These results suggest that this induction of expression may be associated with gastric metaplasia.

Carcinoma cells, such as colonic, prostate, and pancreatic cancer cells [8, 21, 22], reduce their aggressiveness *in vitro* or *in vivo* when forced to express β3Gn-T6. Forced expression of the core 3 structure destabilizes oncoprotein MUC1 [22], affecting downstream signals and upregulating cell cycle inhibitor p21 [22]. The expression of β3Gn-T6 also leads to a reduction in the formation of the α2β1 integrin complex, subsequently reducing the level of phosphorylated FAK relative to total FAK, thereby leading to decreased tumor progression [21, 22]. β3Gn-T6 alters cancer cell invasion through impairment of actin stress fiber organization [21, 37]. Here, we demonstrated clinical significance of β3Gn-T6 expression, which turned out to be a favorable prognostic factor in PDAC, consistent with the known effects of β3Gn-T6 in cancer biology.

In the biosynthesis of *O*-glycans (Fig 1), the processes of formation of core 1 and core 3 structures compete with each other for the same substrate, although the expression levels of T antigen and β3Gn-T6 only weakly correlated in PDAC. This finding suggests that the core 1 structure (T antigen) is mostly modified, i.e., by extension, branching, or sialylation, which are not recognized by PNA. The extended core 1 structure detected by antibody MECA-79, which was only a limited 6-sulfated structure of extended core 1, weakly but statistically significantly negatively correlated with β3Gn-T6 levels (Table 4).

The Tn antigen is one of the representative truncated structures, whose expression is abundant in many types of carcinoma cells owing to the loss of Cosmc, a chaperone for core 1 synthase [9, 38]. Our study revealed that the positivity and Exp-score of the Tn antigen in PDAC cells were high (Table 3 and Fig 3), despite the high expression of sLeX in PDAC cells, suggesting that Cosmc inactivation does not entirely explain the presence of the Tn antigen in our cohort.

This study has several limitations. First, data collection and analyses were performed retrospectively. Second, it was difficult to investigate detailed glycan structural alterations and their changed biosynthesis in cancer cells in clinical samples, because of the lack of antibodies specific for various glycan structures. Therefore, our conclusions are drawn from speculation based on the limited findings. Further studies would be warranted to clarify the molecular mechanism of glycan alterations in PDAC.

In summary, this study is the first to report on the clinicopathological significance of β3Gn-T6 expression in PDAC. β3Gn-T6 was found to be expressed most highly in PDAC cells in differentiated adenocarcinoma and was significantly associated with longer DFS in PDAC patients. Our findings on the molecular mechanisms underlying the induction of core 3 *O*-glycan in PDAC provide a basis for its use as a therapeutic tool.

## Supporting information

S1 Fig Immunohistochemical or lectin-histochemical positive features. Positive staining in immunohistochemical or lectin-histochemical analyses for β3Gn-T6 (B,C), T antigen (staining with PNA) (E,F), Tn antigen (staining with LU-35) (H,I), 6-sulfo *N*-acetyllactosamine on extended core 1 *O*-glycan (staining with MECA-79) (K,L), sLeX (staining with CSLEX1) (N,O), sLeX (staining with HECA-452) (Q,R), sLeX on core 2 *O*-glycan (staining with ST-439) (T,U), and MUC5AC (W,X) was investigated, and their corresponding histological features were revealed by hematoxylin and eosin staining (A, D, G, J, M, P, S, V). The left and the central panels of the photos are a middle-power view, and the right one is a high-power view. (A–C) Cytoplasmic dotlike staining in moderately differentiated adenocarcinoma cells was found by β3Gn-T6 immunohistochemical analysis, whose pattern was consistent with staining in the Golgi apparatus. (D–F) Cytoplasmic staining, especially in the apical portion of adenocarcinoma cells, was found in PNA lectin-histochemical analysis. (G–I) LU-35 staining yielded a cytoplasmic and sometimes membranous pattern, especially on the luminal surface of the carcinoma gland. (J–L) Mainly membranous staining of MECA-79, especially in the luminal surface of NPDEs, was observed in normal pancreatic tissue. (M–O) Cytoplasmic and membranous staining in adenocarcinoma cells was detected by CSLEX1 immunohistochemistry. (P–R) HECA-452 staining yielded both membranous and cytoplasmic patterns. (S–U) ST-439 staining showed positive cytoplasmic and membranous patterns in some cancer cells. (V–X) MUC5AC usually stained in adenocarcinoma cells with clear to light eosinophilic cytoplasm.
(TIF)

S2 Fig Comparison of glycan antigens and β3Gn-T6 expression among histological grades of PDACs (n = 156). (A) β3Gn-T6, (B) T antigen (PNA), (C) Tn antigen (LU-35), (D) 6-sulfo *N*-acetyllactosamine on extended core 1 *O*-glycan (MECA-79), (E) sLeX (CSLEX1), (F) sLeX (HECA-452), and (G) sLeX on core 2 *O*-glycan (ST-439). Boxes represent medians and interquartile ranges. Crosses represent mean values. Whiskers represent the minimum and maximum 1.5 interquartile ranges. Circles represent extremes. Exp-scores were compared and analyzed using the Kruskal–Wallis test followed by Dunn–Bonferroni's post hoc analysis.
(TIF)

S3 Fig Kaplan-Meier survival curves for OS in patients with PDAC according to (A) β3Gn-T6, (B) T antigen (PNA), (C) Tn antigen (LU-35), (D) 6-sulfo *N*-acetyllactosamine on extended core 1 *O*-glycan (MECA-79), (E) sLeX (CSLEX1), (F) sLeX (HECA-452), and (G) sLeX on core 2 *O*-glycan (ST-439). Any antigens are not significantly associated with patient outcome.
(TIF)

S1 Table Primer sequences and Universal Probe Library probes for qRT-PCR. (DOCX)
