# Supplementary figures and images for: Clinicopathological significance of core 3 O-glycan synthetic enzyme, β1,3-N-acetylglucosaminyltransferase 6 in pancreatic ductal adenocarcinoma
Source: PLoS One. 2020 Nov 30;15(11):e0242851. doi: 10.1371/journal.pone.0242851 (PMC7703919; doi:10.1371/journal.pone.0242851)

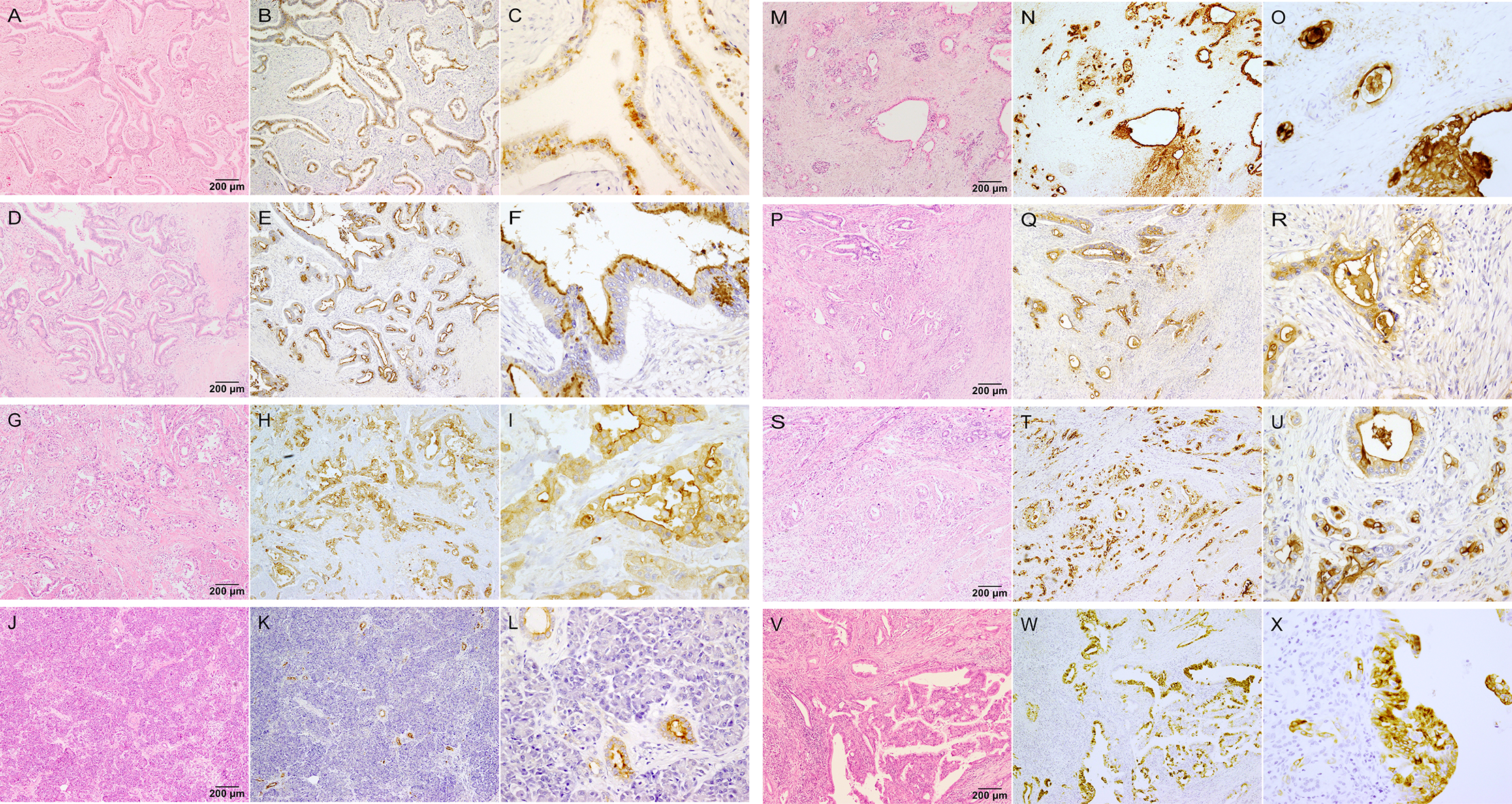

Supplement: S1 Fig — Positive staining in immunohistochemical or lectin-histochemical analyses for β3Gn-T6 (B,C), T antigen (staining with PNA) (E,F), Tn antigen (staining with LU-35) (H,I), 6-sulfo N-acetyllactosamine on extended core 1 O-glycan (staining with MECA-79) (K,L), sLeX (staining with CSLEX1) (N,O), sLeX (staining with HECA-452) (Q,R), sLeX on core 2 O-glycan (staining with ST-439) (T,U), and MUC5AC (W,X) was investigated, and their corresponding histological features were revealed by hematoxylin and eosin staining (A, D, G, J, M, P, S, V). The left and the central panels of the photos are a middle-power view, and the right one is a high-power view. (A–C) Cytoplasmic dotlike staining in moderately differentiated adenocarcinoma cells was found by β3Gn-T6 immunohistochemical analysis, whose pattern was consistent with staining in the Golgi apparatus. (D–F) Cytoplasmic staining, especially in the apical portion of adenocarcinoma cells, was found in PNA lectin-histochemical analysis. (G–I) LU-35 staining yielded a cytoplasmic and sometimes membranous pattern, especially on the luminal surface of the carcinoma gland. (J–L) Mainly membranous staining of MECA-79, especially in the luminal surface of NPDEs, was observed in normal pancreatic tissue. (M–O) Cytoplasmic and membranous staining in adenocarcinoma cells was detected by CSLEX1 immunohistochemistry. (P–R) HECA-452 staining yielded both membranous and cytoplasmic patterns. (S–U) ST-439 staining showed positive cytoplasmic and membranous patterns in some cancer cells. (V–X) MUC5AC usually stained in adenocarcinoma cells with clear to light eosinophilic cytoplasm. (TIF) [file pone.0242851.s001.tif]

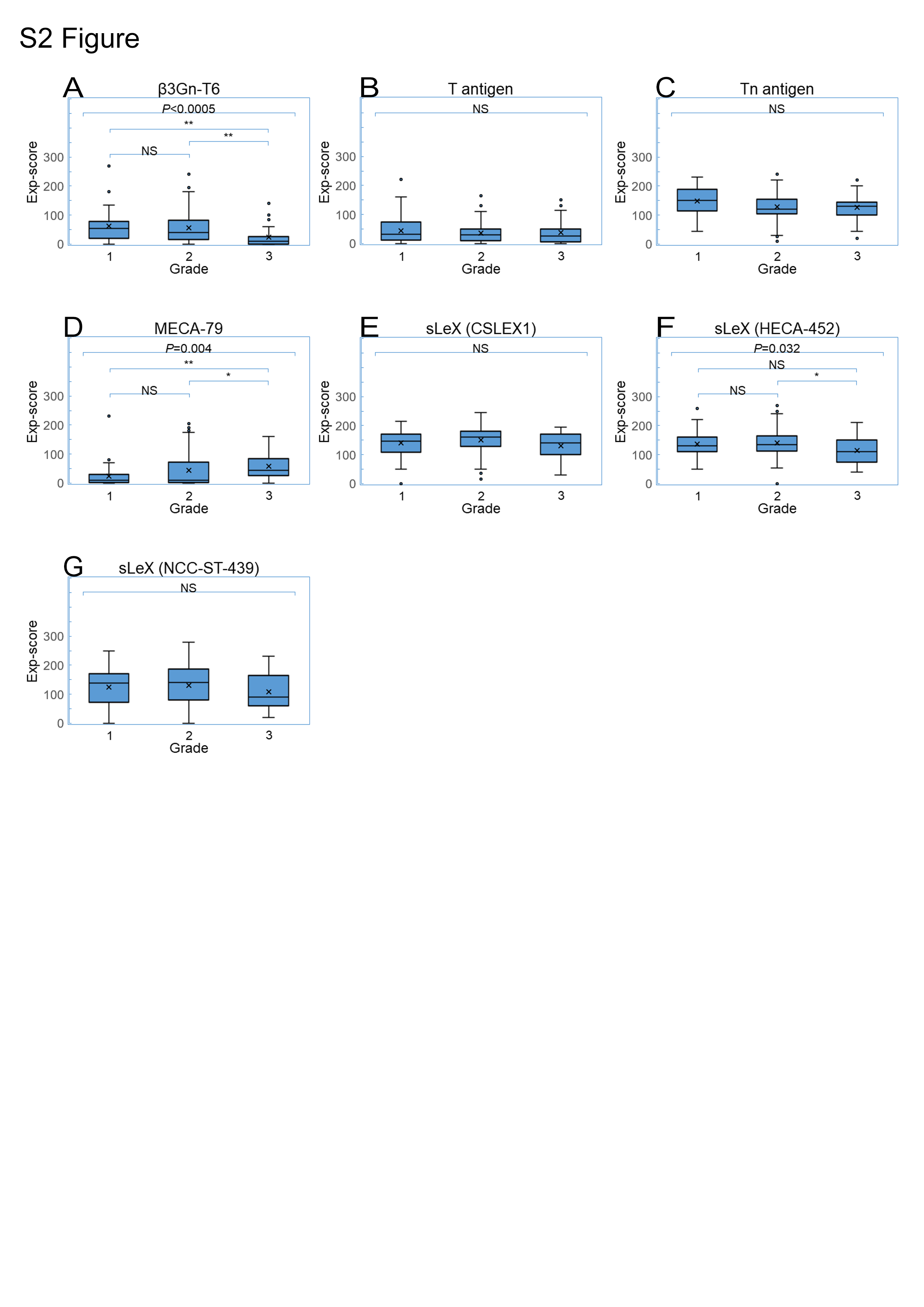

Supplement: S2 Fig — (A) β3Gn-T6, (B) T antigen (PNA), (C) Tn antigen (LU-35), (D) 6-sulfo N-acetyllactosamine on extended core 1 O-glycan (MECA-79), (E) sLeX (CSLEX1), (F) sLeX (HECA-452), and (G) sLeX on core 2 O-glycan (ST-439). Boxes represent medians and interquartile ranges. Crosses represent mean values. Whiskers represent the minimum and maximum 1.5 interquartile ranges. Circles represent extremes. Exp-scores were compared and analyzed using the Kruskal–Wallis test followed by Dunn–Bonferroni's post hoc analysis. (TIF) [file pone.0242851.s002.tif]

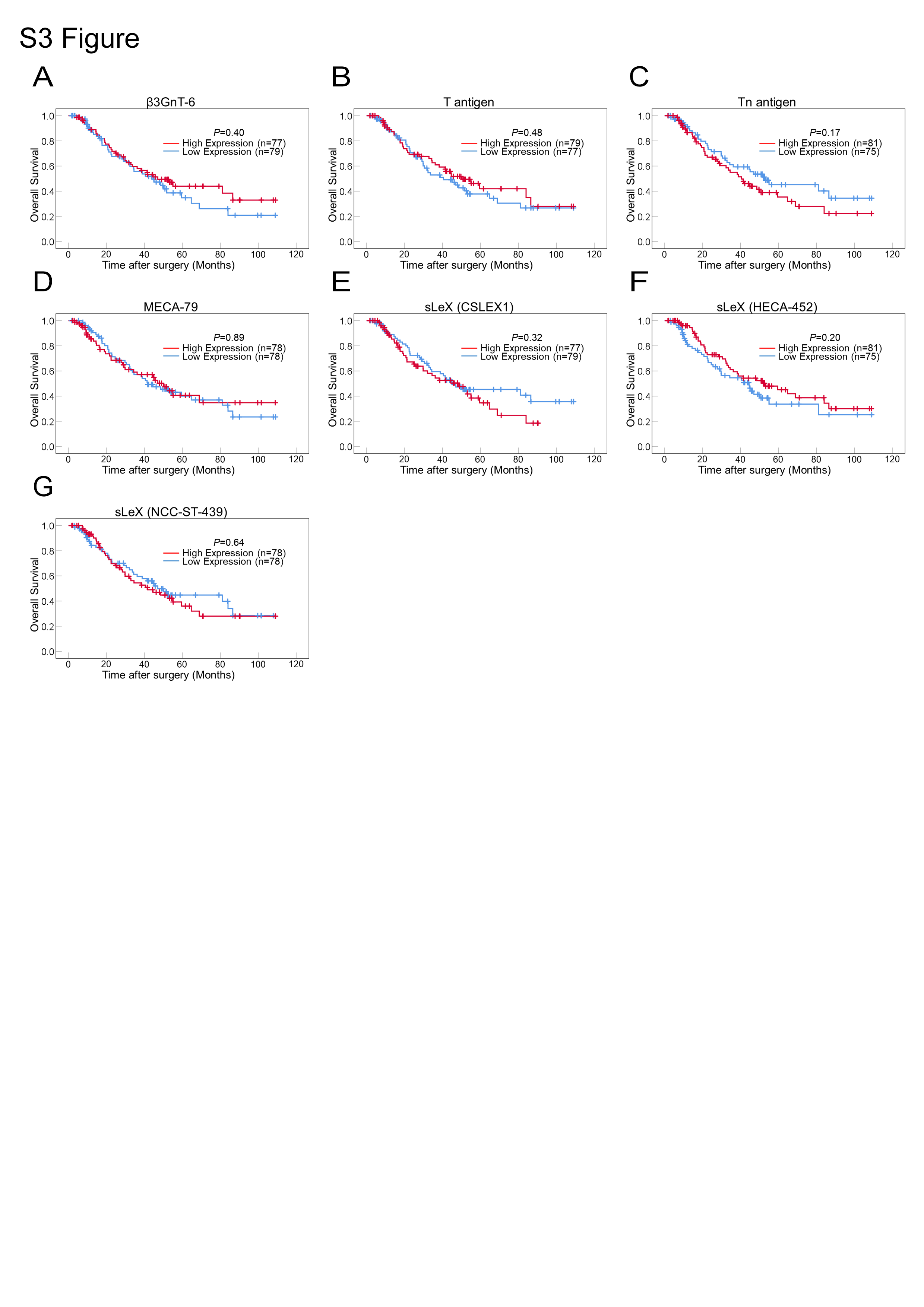

Supplement: S3 Fig — Kaplan-Meier survival curves for OS in patients with PDAC according to (A) β3Gn-T6, (B) T antigen (PNA), (C) Tn antigen (LU-35), (D) 6-sulfo N-acetyllactosamine on extended core 1 O-glycan (MECA-79), (E) sLeX (CSLEX1), (F) sLeX (HECA-452), and (G) sLeX on core 2 O-glycan (ST-439). Any antigens are not significantly associated with patient outcome. (TIF) [file pone.0242851.s003.tif]
