# Supplementary material for: Clinicopathological significance of core 3 O-glycan synthetic enzyme, β1,3-N-acetylglucosaminyltransferase 6 in pancreatic ductal adenocarcinoma
Source: PLoS One. 2020 Nov 30;15(11):e0242851. doi: 10.1371/journal.pone.0242851 (PMC7703919; doi:10.1371/journal.pone.0242851)
Supplement: Supplementary file 2 [file pone.0242851.s004.docx]

S1 Table. Primer sequences and Universal Probe Library probes for quantitive RT-PCR.

Name of genes	Universal Probe Library Probe	Left primer	Right primer
ACTB	#64	ccaaccgcgagaagatga	tccatcacgatgccagtg
GCNT1	#43	cctgacagcatgtgaagtgc	cttgaaatgaagagcagcaca
GCNT3	#25	atccctaagcaggagagaagc	aggtggagaccgagcaca
GCNT4	#77	ctctaatacttagcatagctcctttgg	ggaaccccacgttcagaaa
B3GNT6	#16	gggatggctcctgtctatttc	tgcagtgctaagaaactcacg
MUC5AC	#40	gatcccgacctaccaggag	caggctcgaggagaccac
